# Nano-sized Al_2_O_3_ particle-induced autophagy reduces osteolysis in aseptic loosening of total hip arthroplasty by negative feedback regulation of RANKL expression in fibroblasts

**DOI:** 10.1038/s41419-018-0862-9

**Published:** 2018-08-06

**Authors:** De Li, Chenglong Wang, Zhuokai Li, Hui Wang, Jiye He, Junfeng Zhu, Yuehui Zhang, Chao Shen, Fei Xiao, Yuan Gao, Xiang Zhang, Yang Li, Peng Wang, Jianping Peng, Guiquan Cai, Bin Zuo, Yuehua Yang, Yun Shen, Weidong Song, Xiaoling Zhang, Lei Shen, Xiaodong Chen

**Affiliations:** 1 Department of Orthopedic Surgery, Xin Hua Hospital Affiliated to Shanghai Jiao Tong University School of Medicine, Shanghai, China; 2 Department of Orthopedic Surgery, Sun Yat-Sen memorial hospital affiliated to Sun Yat-Sen university, Guangzhou, China

## Abstract

Aseptic loosening is mainly caused by wear debris generated by friction that can increase the expression of receptor activation of nuclear factor (NF)-κB (RANKL). RANKL has been shown to support the differentiation and maturation of osteoclasts. Although autophagy is a key metabolic pathway for maintaining the metabolic homeostasis of cells, no study has determined whether autophagy induced by Al_2_O_3_ particles is involved in the pathogenesis of aseptic loosening. The aim of this study was to evaluate RANKL levels in patients experiencing aseptic loosening after total hip arthroplasty (THA) and hip osteoarthritis (hOA) and to consequently clarify the relationship between RANKL and LC3II expression. We determined the levels of RANKL and autophagy in fibroblasts treated with Al_2_O_3_ particles in vitro while using shBECN-1 interference lentivirus vectors to block the autophagy pathway and BECN-1 overexpression lentivirus vectors to promote autophagy. We established a novel rat model of femoral head replacement and analyzed the effects of Al_2_O_3_ particles on autophagy levels and RANKL expression in synovial tissues in vivo. The RANKL levels in the revision total hip arthroplasty (rTHA) group were higher than those in the hOA group. In patients with rTHA with a ceramic interface, LC3II expression was high, whereas RANKL expression was low. The in vitro results showed that Al_2_O_3_ particles promoted fibroblast autophagy in a time- and dose-dependent manner and that RANKL expression was negatively correlated with autophagy. The in vivo results further confirmed these findings. Al_2_O_3_ particles induced fibroblast autophagy, which reduced RANKL expression. Decreasing the autophagy level promoted osteolysis and aseptic prosthetic loosening, whereas increasing the autophagy level reversed this trend.

## Introduction

THA is one of the most effective procedures for treating severe trauma, rheumatoid arthritis, osteoarthritis and other end-stage joint diseases^1–3^. Although complications rarely arise after arthroplasty, periprosthetic osteolysis and subsequent aseptic prosthetic loosening are the most common complications that limit the longevity of prostheses^4–6^.

Wear debris that separate from the surface of prostheses is a primary contributor to aseptic loosening^7,8^. Current bearing surface materials for THA have four main combinations including metal-on-polyethylene (MoP), ceramic-on-polyethylene, ceramic-on-ceramic (CoC), and metal-on-metal (MoM), of which MoP implants have been the most widely used for over 40 years. MoP bearing surfaces were once considered the gold standard for THA. However, there have always been concerns about polyethylene wear debris-induced aseptic loosening and osteolysis^9–12^. MoM resurfacings have caused several adverse reactions, such as pseudotumor development, hypersensitivity reactions, and toxicity from metal ions; MoM prostheses were recalled in 2010 due to significantly greater revision rates compared with all other conventional prostheses^13,14^. CoC surface materials also have a long history. As early as April 1970, Dr. Pierre Boutin first introduced ceramic bearings into the field of artificial joints^15^. However, early CoC prostheses had a higher revision rate due to poor designs, inadequate material properties, and imperfect surgical techniques. Therefore, the following complications related to CoC prosthesis have been observed: ceramic fracture, rupture of the prosthesis during operation, liner chipping on insertion, liner canting or dissociation, edge loading, squeaking and aseptic loosening of the prosthesis. Aseptic loosening of CoC bearings during 1970s and 1980s were mainly due to the method of fixation of the components rather than a biological reaction to wear debris^15–18^. Studies in vitro have shown fewer macrophage reactions and decreased cytokine secretion with exposure to ceramic particles in comparison to titanium particles^16,19^. Histological staining has identified ceramic wear particles within individual macrophages in peri-prosthetic tissues, and these particles did not generate serious foreign body granulomas^15^.

Nevertheless, the effects of Al_2_O_3_ particles on aseptic loosening remain unclear. Research has shown that Al_2_O_3_ particles can induce autophagy formation^20^. Our prior studies also support this conclusion; moreover, we have determined that Al_2_O_3_ particles can increase the level of autophagy more than Cobalt-Chromium alloy particles. Therefore, we hypothesized that Al_2_O_3_ particles can induce RANKL expression, resulting in osteoclast formation. Additionally, Al_2_O_3_ particle-induced autophagy reduces osteolysis by negative feedback regulation of RANKL expression in fibroblasts. Nevertheless, the effects of Al_2_O_3_ particles on aseptic loosening remain unclear, particularly with regard to the regulation of autophagy and osteoclasts. Thus, we determined the RANKL and autophagy levels in fibroblasts challenged with Al_2_O_3_ particles in vitro. We also established a novel rat model of femoral head replacement to analyze the effects of Al_2_O_3_ particles on autophagy and RANKL levels in vivo.

The expression of osteoclasts is reportedly increased in the pseudomembrane of the hips after revision arthroplasty^21^. The RANKL, which is a member of the tumor necrosis factor family, facilitates the differentiation and maturation of osteoclasts. The macrophage colony-stimulating factor (M-CSF) is another essential cytokine for osteoclast differentiation and maturation^22–25^. Fibroblasts and macrophages are the primary sources of RANKL. However, no study has clarified how RANKL is regulated in fibroblasts challenged with Al_2_O_3_ particles in patients experiencing aseptic loosening.

Numerous studies have recently demonstrated that autophagy is a dynamic process responsible for the turnover of cellular organelles and long-lived proteins, suggesting that autophagy plays a crucial role in cellular homeostasis and adaptation to adverse environments^20,26–28^. Previous studies have demonstrated that certain nanomaterials induce autophagy to degrade materials that are perceived by cells as foreign or aberrant^20,29,30^. The metal wear particles retrieved from patients experiencing aseptic loosening are reportedly as small as 50 nm (ranging from 6 to 834 nm)^31^. Currently, the relationship between aseptic loosening induced by Al_2_O_3_ wear debris and autophagy remains unclear.

In the present study, we hypothesized that wear-debris-induced autophagy in fibroblasts is responsible for Al_2_O_3_-particle-induced aseptic loosening. Autophagy inhibits the activation of osteoclasts and reduces osteolysis and aseptic loosening. We evaluated our hypothesis by means of synovial samples collected from patients with rTHA and hOA, fibroblasts and a novel rat model of femoral head replacement.

## Results

### Imaging and histomorphological manifestations of aseptic loosening in patients with THA of MoM, MoP, and CoC-bearing surfaces

**Fig. 1:**
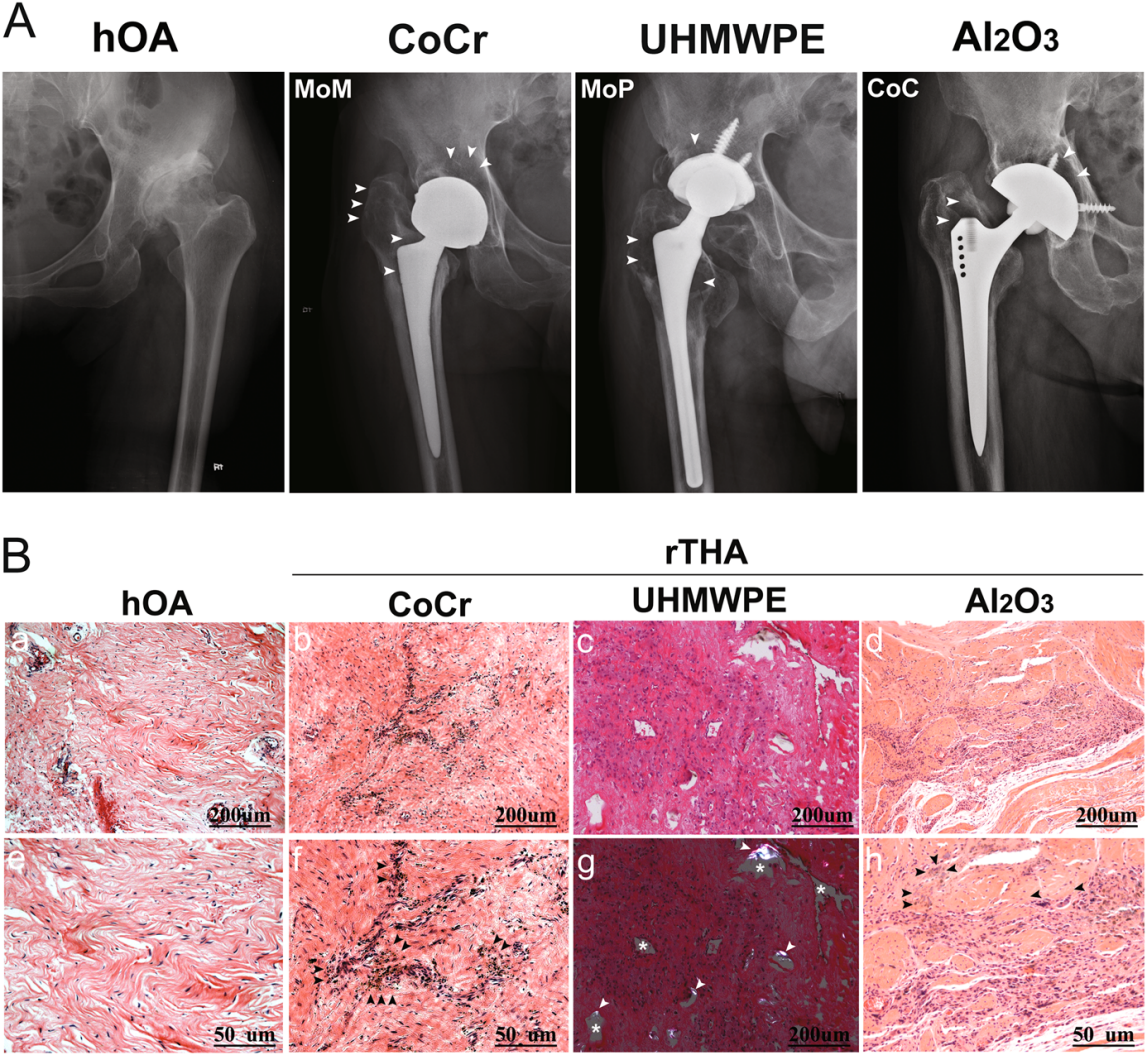
Imaging and histomorphological manifestations of prosthesis loosening with different frictional interfaces. **a** Anterior and posterior X-ray images demonstrating a wide range of osteolytic areas (white arrow) around the proximal femur and the acetabular cup at the MoM, MoP, and CoC interfaces in hOA patients. **b** (a, e) H&E staining showed that inflammatory cells infiltrated into the synovial tissue of patients with hOA. b, f H&E staining of the synovial tissue of the hip joint of patients with rTHA (metal-on-metal interface, MoM) showed a large number of black particles of uniform size (black arrow). c H&E staining of the synovial tissues from the hip joints of patients with rTHA (metal-on-polyethylene interface, MoP) showed that the uneven area of the blank (white asterisk) was surrounded by increased synovial fibroblasts and aggregated mononuclear macrophages in the synovium from patients with rTHA. (g) UHMWPE particles were observed as double refractions by polarized light micrographs (white arrow). d, h Gray or black micro- and nanosized scattered ceramic particles were observed in the synovium with fewer inflammatory cells (black arrow) from patients with rTHA (ceramic-on-ceramic interface, CoC). a, b, c, d, g Scale bar: 200 μm; (e, f, h) Scale bar: 50 μm. Revision total hip arthroplasty (rTHA), hip osteoarthritis (hOA); CoCr, UHMWPE, and Al_2_O_3_ refer to the wear debris of MoM, MoP, and CoC, respectively

Four groups were formed, namely, hOA (control), CoCr, UHMWPE and Al_2_O_3_. For the rTHA group, CoCr, UHMWPE, and Al_2_O_3_ were the wear debris of the MoM, MoP, and CoC interfaces, respectively. The range of the osteolytic areas (white arrow) was determined with anterior and posterior X-ray images. (Fig. 1a). The Al_2_O_3_ group showed a smaller osteolytic area than the CoCr and UHMWPE groups. H&E staining revealed that inflammatory cells infiltrated into the synovial tissue of patients with hOA (Fig. 1b, panels a, e). The rTHA group had more inflammatory cells than the hOA group. Moreover, CoCr (Fig. 1b, panels b, f), UHMWPE (Fig. 1b, panels c, g), and Al_2_O_3_ (Fig. 1b, panels d, h) wear debris were observed in the corresponding groups. However, the Al_2_O_3_ group had fewer synovial fibroblasts and mononuclear macrophages than the CoCr and UHMWPE groups, suggesting that a less intense inflammatory response occurred in the Al_2_O_3_ group (Fig. 1b).

### Upregulation of autophagy induced by wear debris with downregulation of RANKL expression in the synovial tissue of patients with rTHA

**Fig. 2:**
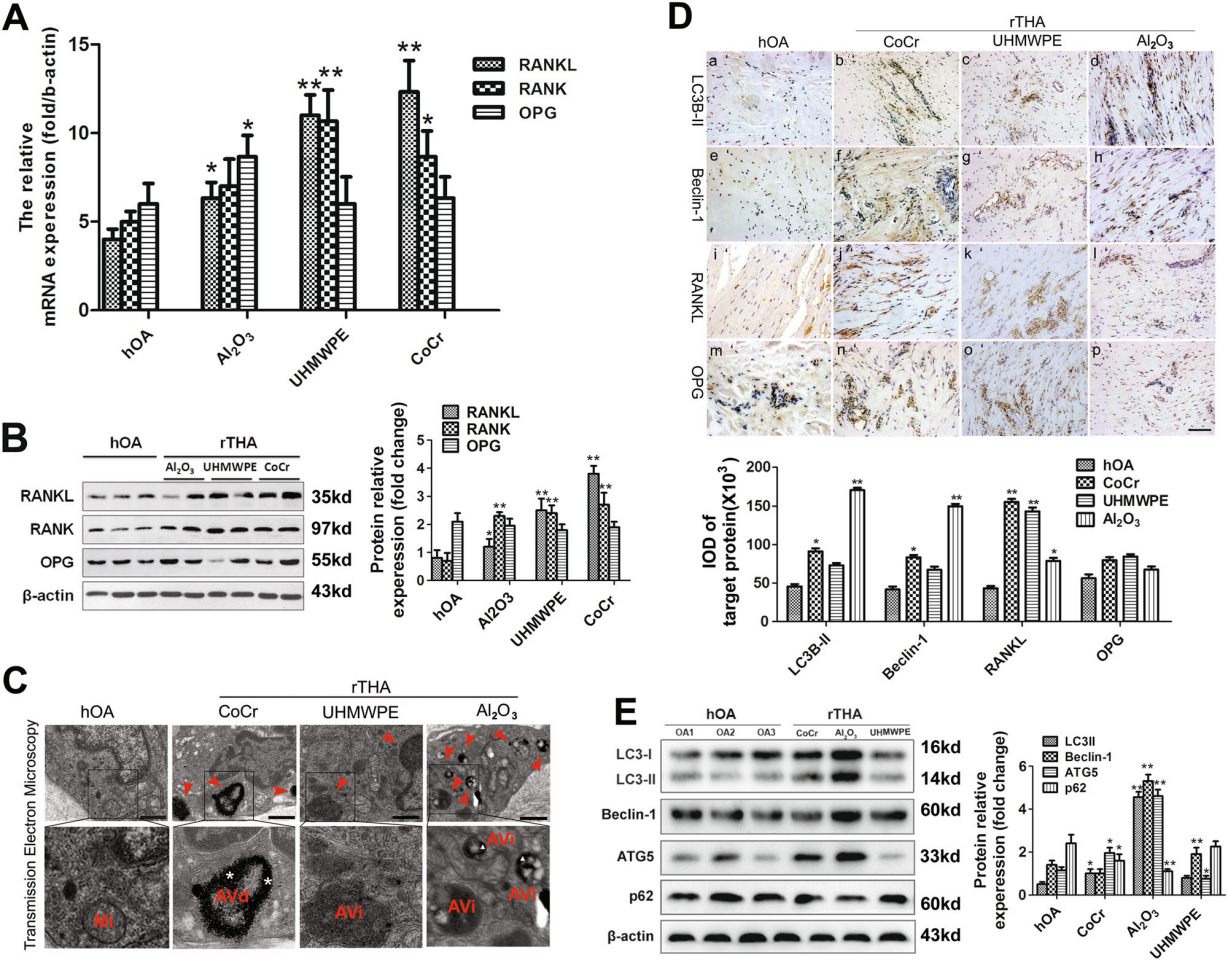
Autophagy induced by wear debris and expression of RANKL, RANK, OPG in the synovial tissue of patients with rTHA. **a**–**b** The RNA and protein expression levels of RANKL, RANK, and OPG in the synovial tissue of patients with different frictional prosthesis loosening. The right side shows the quantitative results of Western blot analysis. **c** Detection of autophagy in the synovial tissues of patients experiencing loosening of prosthetics with different friction interfaces, as revealed by transmission electron microscopy (red arrow). Scale bar: 500 nm. **d** The expression of RANKL, OPG, Beclin1, and LC3B-II in the synovial tissues of patients experiencing prosthetic loosening, as detected by immunohistochemical staining. Scale bar: 100 μm. **e** Western blot analysis was used to detect the expression of autophagy-related proteins in the synovial tissue of each group. The data are expressed as the means ± SD of three independent experiments. [*n* = 3; * compared with the hOA group (*P < 0.05; **P < 0.01).] (*AVi* initial autophagic vacuoles, *AVd* degrading autophagic vacuoles, *MI* mitochondria)

RT-PCR and Western blot were performed to assess the levels of RANKL, RANK, and OPG. The levels in the rTHA group were higher than those in the hOA group. In addition, the protein level of RANKL in the Al_2_O_3_ group was lower than that in the CoCr and UHMWPE groups (*P* < 0.05) (Figs. 2a, b). A transmission electron microscope was used to detect autophagy in the synovial tissue of patients experiencing loosening of prostheses with different friction interfaces. The CoC interface group showed different stages of autophagy formation. Dark black Al_2_O_3_ particles were wrapped in autophagosomes. The typical double-layer structure was observed in the high-power field, and its quantity and volume were markedly higher than that in the MoM and MoP groups (Fig. 2c). The protein expression of RANKL, OPG, Beclin1, and LC3B-II in the synovial tissue of patients experiencing prosthetic loosening was detected by immunohistochemical staining. The results indicated that the expression of RANKL was negatively correlated with that of Beclin-1 and LC3B-II (Fig. 2d). Western blot analysis was used to detect the expression of autophagy-related proteins, such as LC3-I, LC3-II, Beclin-1, ATG-5, and p62, in the synovial tissue of each group. The Al_2_O_3_ group had a significantly higher expression of autophagy-related proteins than the control, CoCr and UHMWPE groups (Fig. 2e)

### Al_2_O_3_ particles induced autophagy of FLSs

**Fig. 3:**
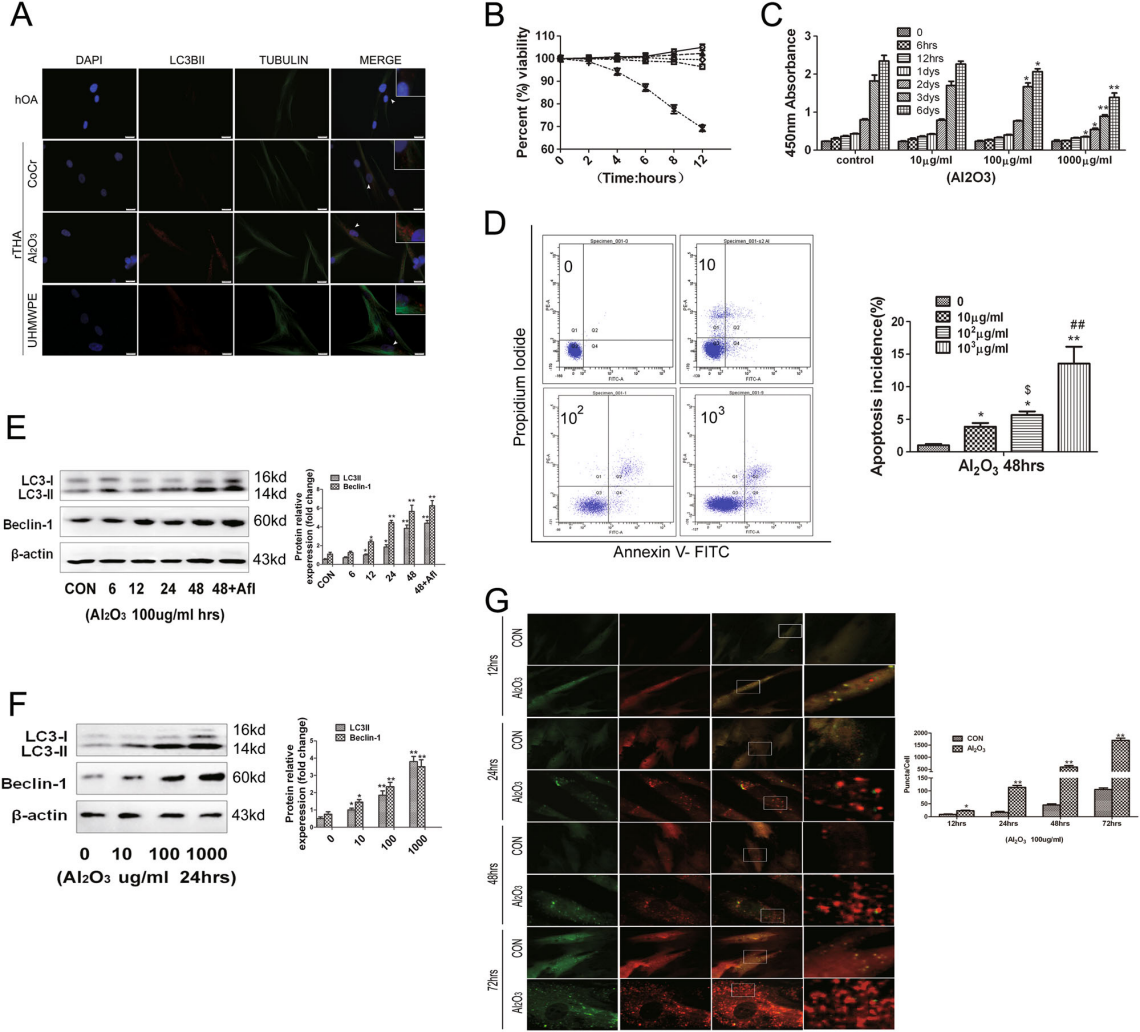
Al_2_O_3_ particles induced autophagy in FLSs. **a** The fluorescence intensity indicating LC3B-II protein levels was observed by immunofluorescence staining: nucleus (DAPI, blue), LC3B-II (red), and Tubulin (green). Scale bar: 25 μm. **b** Lactate dehydrogenase cytotoxicity assay was used to determine the toxicity of Al_2_O_3_ nanoparticles in fibroblasts. **c** Effects of CCK-8 on the proliferation of fibroblast-like synovial cells by Al_2_O_3_ nanoparticles. **d** Effects of Al_2_O_3_ nanoparticles on the apoptosis of fibroblast-like synovial cells by flow cytometry. **e** Western blot analysis showed that the autophagy level of fibroblasts loaded with 100 μg/mL Al_2_O_3_ nanoparticles was increased in a time-dependent manner. **f** Western blot analysis showed that the effect of Al_2_O_3_ nanoparticles at 0, 10, 100, and 1000 μg/ml on the autophagy level in fibroblasts was increased in a dose-dependent manner through 24 h. **g** mRFP-GFP-LC3 autophagy fluorescent double-labeled dynamic observation of the effects of a 100 μg/mL concentration of Al_2_O_3_ nanoparticles on fibroblast autophagy levels; autophagy increased in a time-dependent manner through 72 h. The data are expressed as the means ± SD of three independent experiments. Scale bar: 10 μm. [*n* = 3; * compared with the control group (*P < 0.05; **P < 0.01) ^$^ compared with the control group 10 μg/mL (^$^*P* < 0.05), ^#^ compared with the control group 10 μg/mL (^#^*P* < 0.05; ^##^*P* < 0.01)]

The fluorescence intensity level indicating the amount of the LC3B-II protein was observed by immunofluorescence staining. The Al_2_O_3_ group showed a higher fluorescence intensity than the other experimental groups (Fig. 3a). LDH cytotoxicity testing revealed that the groups treated with Al_2_O_3_ concentrations of 10, 100, and 1000 μg/mL had no significant difference from the control group within 12 h (Fig. 3b). CCK-8 was used to analyze the proliferation of FLSs by Al_2_O_3_, and the results showed a significant difference between the concentration at 100 μg/mL Al_2_O_3_ and the control group within 3 d. The difference became more marked at 1000 μg/mL on the following day. Therefore, 100 μg/mL Al_2_O_3_ was used in subsequent experiments (Fig. 3c). The effects of Al_2_O_3_ nanoparticles on the apoptosis of FLSs were detected by flow cytometry. The results indicated that the number of apoptotic cells was low at a concentration of 100 μg/ml, and the number of apoptotic cells increased significantly when the concentration was more than 1000 μg/mL (Fig. 3d). Western blot analysis suggested that the autophagy level of fibroblasts treated with 100 μg/mL Al_2_O_3_ particles increased in a time-dependent manner (Fig. 3e). In addition, the Western blot results demonstrated that the effect of Al_2_O_3_ nanoparticles on the autophagy level in fibroblasts increased in a dose-dependent manner (Fig. 3f). MRFP-GFP-LC3 autophagy fluorescent double-labeled dynamic observation revealed that the effects of concentration of 100 μg/mL Al_2_O_3_ nanoparticles on fibroblast autophagy levels was time-dependent within 72 h (Fig. 3g).

### Autophagy induced by Al_2_O_3_ nanoparticles attenuated the expression of RANKL in FLSs

**Fig. 4:**
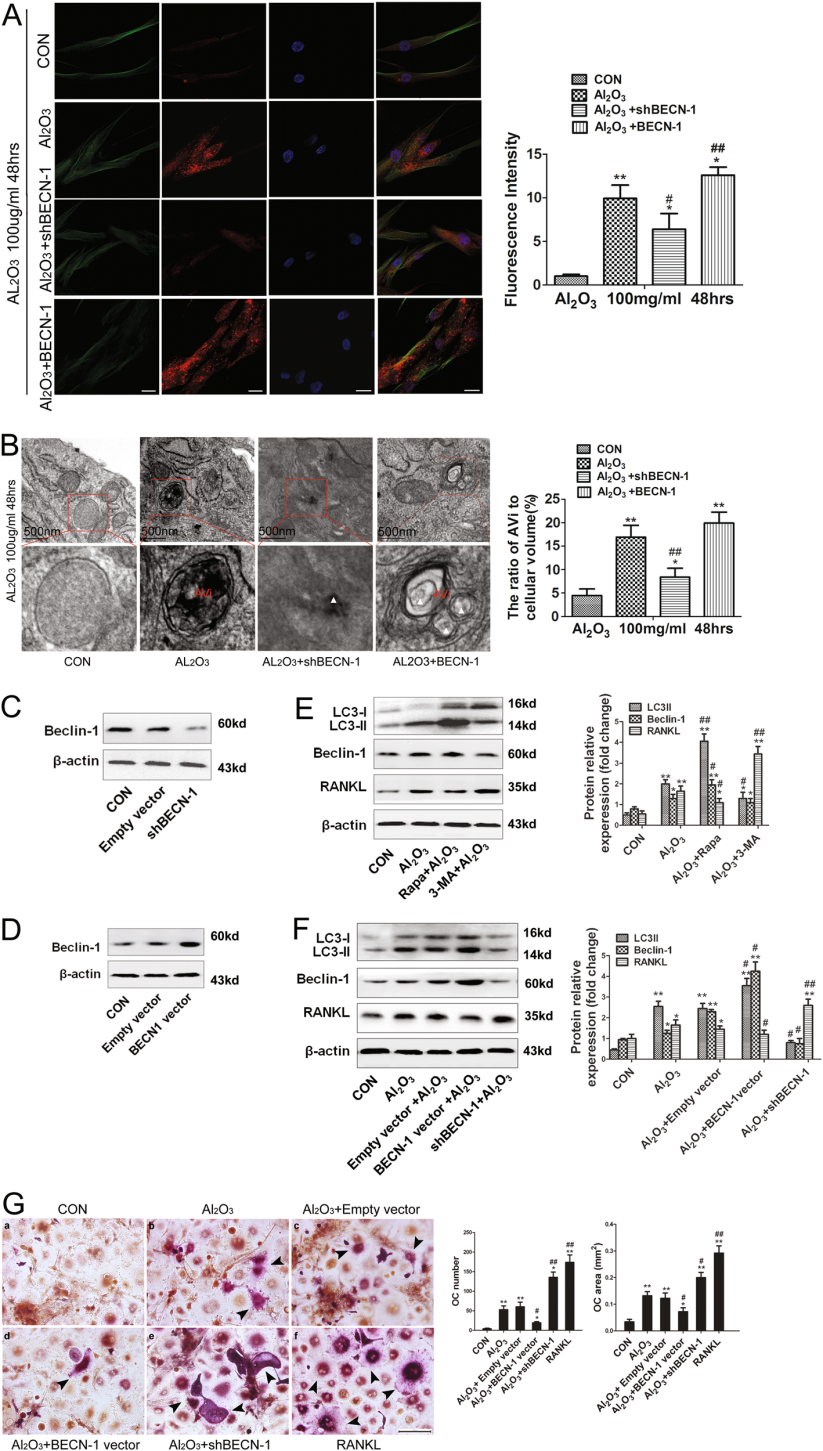
Autophagy induced by Al_2_O_3_ particles mediated the expression of RANKL in FLSs **a** The fluorescence intensity indicating the level of LC3B-II protein was observed by immunofluorescence staining. The fibroblast-like synoviocytes were incubated with 100 μg/ml Al_2_O_3_ nanoparticles for 48 h; Nucleus (DAPI, blue), LC3B-II (red), and Tubulin (green). The experimental groups were as follows: control group, Al_2_O_3_ nanoparticle group, Al_2_O_3_ nanoparticle + BECN-1 group, and Al_2_O_3_ nanoparticle + shBECN-1 group. Scale bar: 25 μm. **b** The formation of autophagosomes in synovial tissues of fibrous synovial cells was observed by transmission electron microscopy, Scale bar: 500 nm; **c**–**d** Western blot analysis confirmed the effectiveness of shBECN-1 and BECN-1 lentiviral vectors; **e**–**f** Western blot analysis was used to detect the effect on the expression of RANKL of the level of autophagy in fibroblasts induced by Al_2_O_3_ nanoparticles, Autophagy Inhibitors, (3-Methyladenine, 3-MA), and Autophagy Inducers (Rapamycin, RAPA); **g** TRAP staining was used to observe the effects of exocrine RANKL on the number and area of osteoclast formation when fibroblast autophagy levels were changed. CM refers to conditioned medium (80% culture medium containing 25 ng/ml M-CSF), RANKL concentrations were 50 ng/ml, Al_2_O_3_ nanoparticle concentrations were 100 μg/ml. The multinucleated giant cells (black arrows) stained with purple were TRAP^+^ cells. Data are expressed as the means ± SD of three independent experiments. Scale bar: 50 μm. [* compared with Control group (*P < 0.05; **P < 0.01). ^#^ compared with Empty vector (^#^*P* < 0.05; ^##^*P* < 0.01)]

The samples were divided into four groups: a control group, an Al_2_O_3_ nanoparticle group, an Al_2_O_3_ nanoparticle + BECN-1 group, and an Al_2_O_3_ nanoparticle + shBECN-1 group. The fluorescence intensity of the LC3B-II protein was observed by immunofluorescence staining. The FLSs were incubated with 100 μg/mL Al_2_O_3_ nanoparticles for 48 h. The results showed that the autophagy level increased in the Al_2_O_3_ nanoparticles compared with control group, while it decreased in the Al_2_O_3_ + shBECN-1 group. This trend was reversed in the Al_2_O_3_ + BECN-1 group (Fig. 4a). Transmission electron microscopy revealed the formation of autophagosomes in the synovial tissue of fibrous synovial cells. The number of autophagosomes in the Al_2_O_3_ nanoparticle group were significantly higher than those in the control and Al_2_O_3_ nanoparticles + shBECN-1 groups (Fig. 4b). Western blot analysis confirmed the effectiveness of the shBECN-1 and BECN-1 lentiviral vectors. Compared with the control and empty vector groups, the shBECN-1 group showed a lower protein level, whereas the BECN-1 lentiviral vector group showed a higher protein level (Figs. 4c, d). Histograms showed that the autophagy levels in fibroblasts were inversely proportional to their intracellular RANKL expression. [autophagy inhibitors (3-methyladenine, 3-MA); autophagic inducers (rapamycin, RAPA)] (Figs. 4e, f). TRAP staining was used to observe the effects of exocrine RANKL on the number and area of osteoclast formation when fibroblast autophagy levels were changed. Compared with the other experimental groups, the shBECN-1 group showed a greater number and a larger area of osteoclast formation, whereas the BECN-1 lentiviral vector group displayed the opposite trend (Fig. 4g).

### A rat model of biologic femoral head replacement

**Fig. 5:**
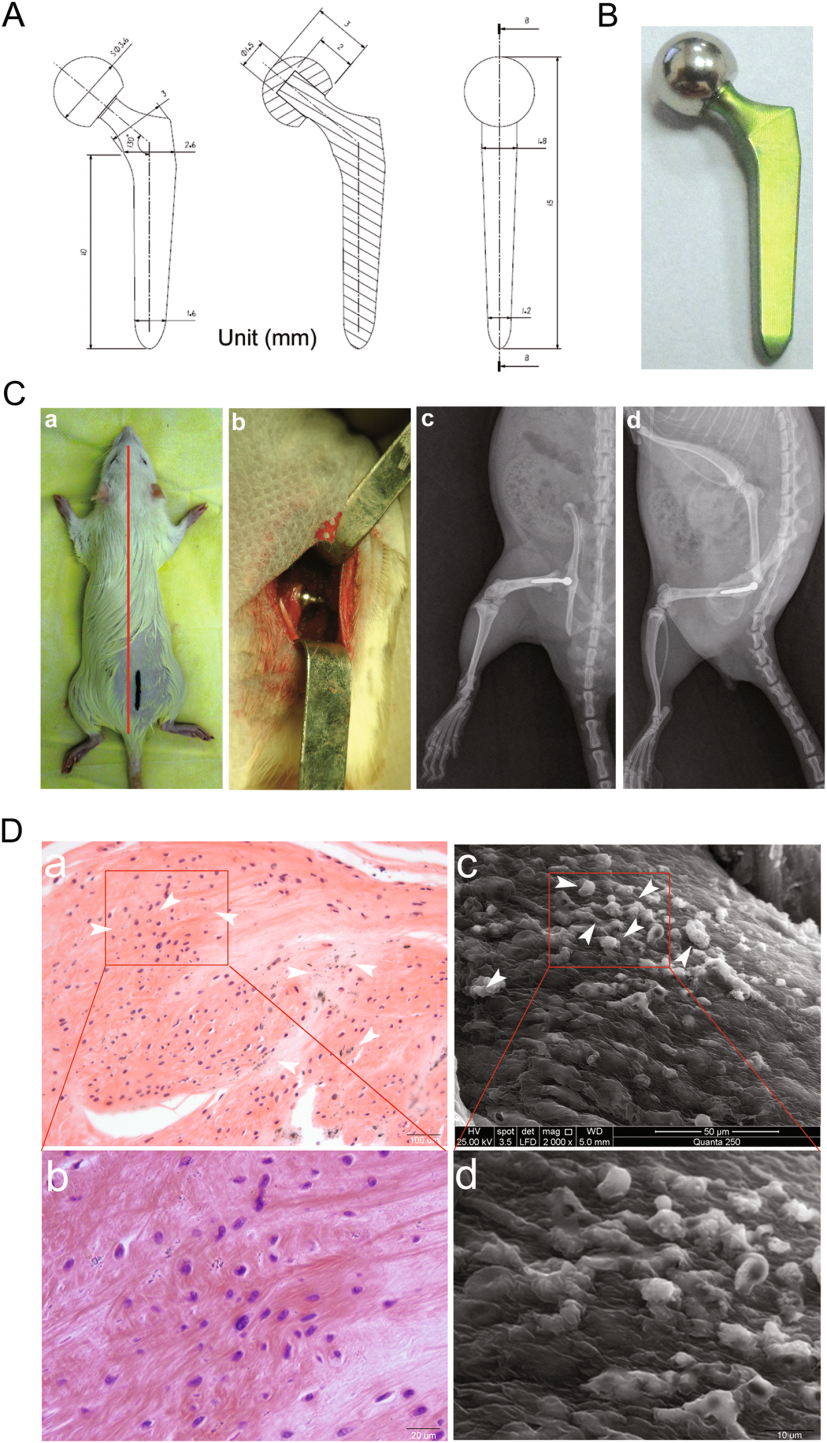
A rat model of biological femoral head replacement **a** CAD drawings of the femoral head prosthesis strategy in rats; **b** The schematic and physical objects of the femoral head prosthesis. **c** Establishing a rat model of biologic femoral head replacement. a Surgical approach, (b) Rat biological femoral head replacement, (c, d) AP & LAT X-ray performance of rat biological femoral head replacement one month after surgery. **d** 4 weeks after biologic femoral head replacement in rats, the morphology of the synovium around the prosthesis was detected. a, b The synovial tissue around the prosthesis was stained with H&E, showing that gray nanoparticles (white arrows) were scattered. c, d The synovial tissue around the prosthesis was examined by scanning electron microscopy, showing the surface of Al_2_O_3_ nanoparticles. Data are expressed as the means ± SD of three independent experiments. (a, Scale bar: 100 μm; c, Scale bar: 50 μm.)

CAD drawings of the femoral head prosthesis in rats are shown in Fig. 5a. A rat model of biologic femoral head replacement is presented in Figs. 5b, c (panel a) shows the surgical approach, (panel b) illustrates the rat biologic femoral head replacement, and (panel c) presents the AP & LAT X-ray performance of rat femoral head replacement one month after surgery. The results suggested that the prosthesis was positioned well. The morphology of the synovial tissue around the prosthesis was measured 4 weeks after the biologic femoral head replacement in rats. The synovial tissue around the prosthesis was stained with H&E. The results revealed that gray Al_2_O_3_ nanoparticles (white arrows) were scattered and that the number of FLSs was greater than that of the control group (Fig. 5d, panels a, b). Scanning electron microscopy of the synovial tissue around the prosthesis showed Al_2_O_3_ nanoparticles on the surface of the synovial tissue (Fig. 5d, panels c, d).

### Autophagy mediated periprosthetic osteolysis by regulating osteoclastogenesis in the rat model of biologic femoral head replacement

**Fig. 6:**
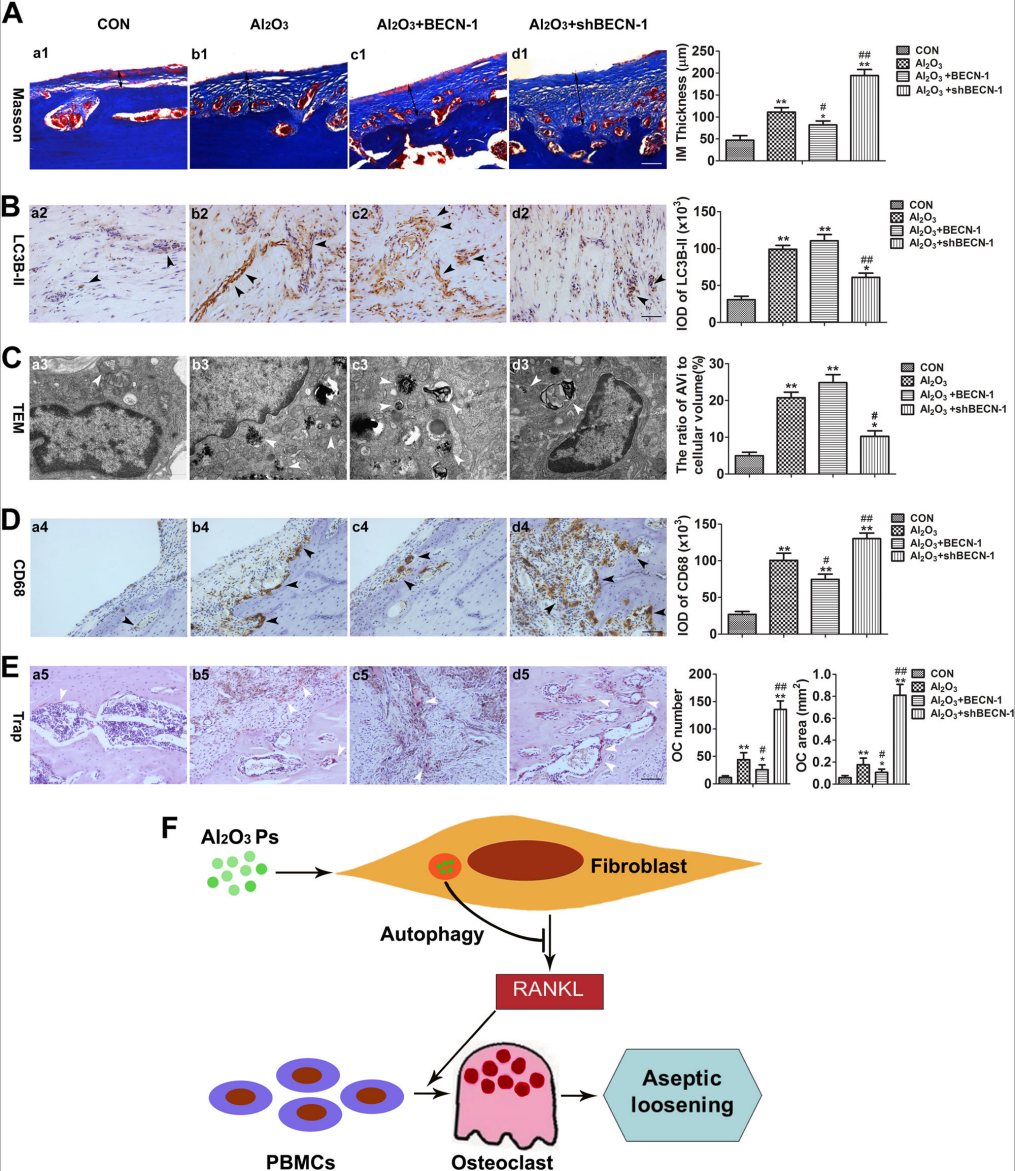
Autophagy mediated periprosthetic osteolysis in the rat model of biological femoral head replacement. **a** Masson’s trichrome staining showed the effects of autophagy levels on synovial tissue thickness around the prosthesis (IM: interface membrane). Scale bar: 50 μm. **b** The expression of LC3B-II in the synovial tissue of patients experiencing prosthetic loosening, as detected by immunohistochemical staining. Scale bar: 50 μm. **c** The synovial tissue around the prosthesis was observed by transmission electron microscopy, showing that Al_2_O_3_ nanoparticles within fibroblast-like synovial cells induced the formation of different stages of autophagy. Scale bar: 500 nm. **d** Immunohistochemical staining showed that CD68 expression was affected by the level of autophagy in the synovial tissue surrounding the prosthesis. Scale bar: 50 μm. **e** TRAP staining to observe the effects of autophagy levels in the synovial tissues around the prosthesis on the number and area of osteoclast formation. Scale bar: 50 μm. **f** Schematic representation of relevant mechanisms indicating the protective role of autophagy induced by Al_2_O_3_ nanoparticles in preventing aseptic loosening. Data are expressed as the means ± SD of three independent experiments. [* compared with Control group (*P < 0.05; **P < 0.01). ^#^ compared with Al_2_O_3_ + Empty vector group (^#^*P* < 0.05; ^##^*P* < 0.01)]

Compared with Al_2_O_3_ nanoparticle group, the Al_2_O3 nanoparticle + BECN-1 group had thinner synovial tissues, as detected by Masson’s trichrome staining; these tissues were thicker in the Al_2_O_3_ nanoparticle + shBECN-1 group (Fig. 6a). The fluorescence intensity indicating the relative amount of LC3B-II protein was observed by immunofluorescence staining. The Al_2_O_3_ group and the Al_2_O_3_ nanoparticle + BECN-1 group showed a higher fluorescence intensity than the Al_2_O_3_ nanoparticle + shBECN-1 group (Fig. 6b). Transmission electron microscopy showed more autophagosomes were induced in the Al_2_O_3_ nanoparticle group and the Al_2_O_3_ nanoparticle + BECN-1 group, whereas the Al_2_O_3_ nanoparticle + shBECN-1 group showed a lower expression (Fig. 6c). Immunohistochemical staining implied that CD68 expression was affected by the autophagy level in the synovial tissue surrounding the prosthesis. Compared with the Al_2_O_3_ nanoparticle group, the Al_2_O3 nanoparticle + shBECN-1 group exhibited greater CD68 expression, whereas the Al_2_O_3_ nanoparticle + BECN-1 group showed reduced expression. This result suggested that, compared with Al_2_O_3_ nanoparticle group, the Al_2_O3 nanoparticle + BECN-1 group had fewer macrophages whereas the Al_2_O_3_ nanoparticle + shBECN-1 group had more (Fig. 6d). TRAP staining was used to observe the effects of autophagy levels on the number and area of osteoclasts in the synovial tissue surrounding the prosthesis. The results showed that the autophagy level was inversely proportional to the number and area of osteoclasts in the synovial tissue surrounding the prosthesis (Fig. 6e). Schematic representation of the involved mechanisms illustrating the protective role of autophagy induced by Al_2_O_3_ nanoparticles in preventing aseptic loosening (Fig. 6f).

## Discussion

Aseptic loosening is the second-most serious joint surgery complication, after periprosthetic osteolysis and is the most significant limitation of THA^32^. The pathogenesis of aseptic loosening of joint prostheses primarily includes interface membranes regarded as foreign bodies by the patient’s body. These debris particles from the prosthesis initiate a reaction that plays a major role in periprosthetic osteolysis and aseptic loosening^33^. Furthermore, wear particles enter into the periprosthetic tissue and are phagocytosed by macrophages, consequently inducing various important events, including cytokine production and osteoclast activation^34^. However, fibroblasts, which constitute 70% of the cells in the pseudosynovial membrane, have received much less research attention than macrophages, which constitute only 15% of the cells in this membrane. Fibroblasts secrete RANKL and promote osteoclast formation when stimulated by titanium particles^35^. Several studies have highlighted the importance of RANKL in osteolysis^7,36,37^.

To date, limited information has been reported regarding the relationship between autophagy, RANKL, and osteolysis, particularly under conditions where Al_2_O_3_ particles surround the hip joints. To gain insight into the mechanism underlying the progress of aseptic loosening caused by Al_2_O_3_ particles, the effect of Al_2_O_3_ particles on autophagy must be investigated. Then, the relationship between autophagy and osteoclasts can be assessed to reveal the role of RANKL in osteolysis and aseptic loosening.

To ascertain whether Al_2_O_3_ particles induced autophagy, we collected serum, synovial fluid, and synovial tissue samples from patients with hOA and rTHA. We then identified fibroblasts and determined the levels of autophagy related-genes, such as LC3II, BECN-1, ATG5, and P62. Higher expression levels of autophagy related-genes were found in the rTHA group compared to the hOA group. A similar trend was observed when the CoC group was compared with the MoM and MoP groups. Numerous studies have reported that autophagy regulates RANKL-induced osteoclast^38–40^, which is consistent with our findings. Higher autophagy levels were observed in the Al_2_O_3_ group, and these levels increased in a time and dose-dependent manner. In contrast, a smaller number and area of osteoclasts was observed in the CoCr and UHMWPE groups. When shBECN-1 interference and BECN-1 overexpression lentiviral vectors were used to block the autophagy pathway and promote autophagy, respectively, the RANKL-induced osteoclast formation showed an opposite trend. We established in vivo a novel rat model of hip replacement and determined the effects of Al_2_O_3_ particles on autophagy levels and RANKL expression in the synovial tissue. We assessed the effects of autophagy on RANKL-induced osteoclasts when autophagy was blocked or increased. Accordingly, the in vivo findings were entirely consistent with our in vitro results.

With regard to the relationship between RANKL and osteolysis followed by aseptic loosening, many studies have verified that functional osteoclasts induced by proinflammatory cytokines result in local osteolysis^41–43^. We identified the same trend in the present study.

Compared with the control group, Al_2_O_3_ particles increased the level of autophagy in the synovial tissue and reduced the RANKL expression. The number and area of osteoclasts in the bone–prosthesis interface tissue increased when the autophagy pathway was blocked. This phenomenon is summarized in our schematic representation of the mechanisms.

Overall, our findings demonstrated that RANKL expression in the serum, synovial fluid, and synovial tissues of rTHA patients was higher than in the hOA group and that Al_2_O_3_ particles induced fibroblast autophagy to reduce RANKL expression. Decreasing the autophagy level promoted osteolysis and the aseptic loosening of prosthesis, whereas increasing the autophagy level inhibited this trend. Our findings suggest that, to some extent, ceramic prostheses performed better than mental and polyethylene prostheses, particularly with regards to osteolysis and aseptic loosening.

In summary, we report the first in vivo study using a femoral head replacement model in rats to characterize particle-induced osteolysis. The results of this study demonstrate that nanosized Al_2_O_3_ particle-induced autophagy attenuates osteolysis in aseptic loosening by negative feedback, reducing the RANKL expression in fibroblasts. This finding indicates that autophagy may be a therapeutic candidate for the prevention and treatment of wear debris-induced osteolysis by inhibiting osteoclast formation and activation. In addition, COC-bearing surfaces may be good choices for younger, more active patients who will need to use their prosthetic joints more over the long term.

## Materials and methods

### Reagents

Rapamycin (R117), Bafilomycin A1 (B1793), and 3-MA (M9281) were purchased from Sigma-Aldrich (St. Louis, MO, USA). Dulbecco’s modified Eagle’s media (DMEM): nutrient mixture F-12 (12400–024) and fetal calf serum (FCS) (10099–141) were obtained from Gibco (Grand Island, NY, USA). RIPA lysis buffer (P0013C) and protease inhibitor cocktail for general use (P1005) were acquired from Beyotime (Haimen, China). Recombinant M-CSF (400–23) and Recombinant RANKL (400–30) were obtained from PeproTech (Rocky Hill, NJ, USA). Beclin1-shRNA (LV-Beclin1-shRNA), Beclin1 (LV-Beclin1), and control (LV-control) lentiviral vector constructs were generated and produced by GenePharma (Shanghai, China). Polyclonal antibodies against beta-actin (β-actin), CD68, beclin1, LC3II/I, LC3B-II, ATG5, P62, RANKL, OPG, and Vimentin were purchased from Novus Biologicals (Littleton, CO, USA).

### Particle preparation

The aluminum particles, which were purchased from Sigma Aldrich, had a mean diameter of less than 50 nm. The samples were soaked separately in ddH2O, anhydrous alcohol, and 70% alcohol for 24 h and then washed with ddH2O three times. Then, the particles were autoclaved for 30 min at 121 °C and 102.9 kPa. Bacterial endotoxin levels (<0.03 EU/mL) were not detected via limulus amoebocyte lysate assay (Sigma-Aldrich, USA). The particles were suspended in phosphate-buffered saline (PBS) at a concentration of 100 mg/mL and refrigerated as stock solutions at 4 °C. For the in vitro and vivo experiments, the particles were further diluted in DMEM/F-12 medium or PBS to attain various concentrations ranging from 10 µg/mL to 1000 µg/mL and ultrasonicated for 20 min prior to their exposure to the cells.

### Specimens and patients

Synovial tissues were collected from the hip joints of patients experiencing aseptic loosening after rTHA. Control specimens were obtained from patients with hOA who underwent primary THA operations. The surgeries were performed from 2013 to 2015 in the orthopedic department of Xinhua Hospital, which was affiliated with the Shanghai Jiao Tong University School of Medicine. This study was approved by the ethics committee of Xinhua Hospital. The samples obtained during the operations were prepared only for testing purposes and with signed informed consent from the patients.

### Cell cultures and treatments

FLSs were isolated from the synovium of the hip joint of patients with aseptic loosening and hOA during their surgeries. The adipose tissues were removed. Then, the synovial specimens were cut into 1–2 mm^3^ pieces and repeatedly washed with PBS. The pieces were digested in DMEM-F12 containing 0.25% trypsinethylenediaminetetraacetic acid (catalog number 25300–054; Gibco/Life Technologies, USA) for 1 h and incubated in a digestion mixture containing 0.1% collagenase I (1 mg/mL; Sigma-Aldrich, USA) for 3 h at 37 °C. The suspension was centrifuged at 200xg for 5 min and cultured in DMEM/F-12 medium with 10% FCS and 1% penicillin–streptomycin at 37 °C under 95% air humidity and 5% CO_2_. The primary human FLSs were obtained and passaged in a six-well plate at a density of 4 × 10^5^ cells. Cells of low passage (P3–P6) were used for further studies according to our previously reported method^44^.

### Lactate dehydrogenase assay

The FLSs were plated in 96-well culture plates at a density of 6 × 103 cells/well and incubated with various concentrations of aluminum particles (0, 10, 100, and 1,000 µg/mL) and puromycin (1 µg/mL) for 12 h. Lactate dehydrogenase (LDH) activity was measured by using the LDH Release Assay Kit (catalog number C0016; Beyotime, China) in accordance with the manufacturer’s instructions.

### CCK-8 assay for cell viability

The cells were seeded in 96-well plates at a density of 6 × 10^3^ cells/well and co-cultured with various concentrations of aluminum particles (0, 10, 100, and 1,000 µg/mL) for 0, 6 h, 12 h, 1, 2, 3, and 6 d. Each specified well was then supplemented with 2 µL of 2-(2-methoxy-4-nitrophenyl)-3-(4-nitrophenyl)-5 -(2,4-disulfo-phenyl)- 2Htetrazolium (WST-8) using a Cell Counting Kit-8 (CCK-8; catalog number C0037; Beyotime, China). The cells were incubated at 37 °C for 2 h. The absorbance of the optical densities at each time point was determined at 450 nm by using a microplate spectrophotometer (Thermo Scientific, Multiskan FC, Waltham, MA, USA).

### Flow cytometry analysis

The FLSs were seeded into six-well plates at a density of 1 × 10^6^ cells/well and cultured with various concentrations of aluminum particles (0, 10, 100, and 1000 µg/mL) for 24 h. After treatment, the cells were stained using the Annexin V-FITC/PI apoptosis detection kit (Invitrogen, USA) in accordance with to the manufacturer’s instructions. The samples were then analyzed by flow cytometry (Beckman Coulter, Miami, FL, USA).

### Cell interfering experiment

LV-Beclin1-shRNA, LV-Beclin1, and nontargeting LV-control constructs were generated and produced by GenePharma (Shanghai, China). The FLSs were seeded into 12-well plates at a density of 1 × 10^5^ cells/well overnight. The complete medium was mixed with Polybrene (Sigma-Aldrich, USA) at a final concentration of 5 µg/mL. The lentiviral vector stock was adjusted to 40 MOI in 500 µL of culture medium. After being cultured overnight, the medium was replaced with fresh medium, and the cells were further cultured for 3–5 d. The positive, stable transfectants were selected using puromycin at a concentration of 2 µg/mL and expanded for further analysis.

### Histology and immunohistochemistry analysis

The synovial tissues of the hip joint, which were obtained during rTHA and THA operations, were fixed with 4% paraformaldehyde for 24 h. After 4, 8, and 12 weeks, the femurs and hips from each group of Sprague-Dawley (SD) rats were fixed and decalcified for 8 weeks at 4 °C. The tissues were embedded in paraffin and were cut in the coronal or sagittal plane centered over the area of particle erosion. Some of the sections were stained with H&E or Masson’s trichrome, and other sections were immunostained with the respective antibodies of interest. Negative controls were obtained without a specific antibody. The specimens were then observed and imaged under a light microscope (Olympus BX-FLA, Japan).

### Immunofluorescence staining

The FLSs were seeded into 24-well plates at a density of 6 × 10^3^ cells/well with sterile glass cover slips. Aluminum particles were added into the well at a concentration of 100 µg/mL for 48 h. After being incubated according to the study design, the cells were fixed with 4% paraformaldehyde for 15 min, blocked with 5% bovine serum albumin (BSA) for 15 min at room temperature, and incubated with Map1-LC3B2 anti-body (NBP1-99115; Novus, USA) in 5% BSA (dilution 1:50) at 4 °C overnight. The treated cells were washed with PBS three times and incubated with a secondary antibody (dilution 1:200), Alexa Fluor 555 donkey anti-mouse IgG (catalog number A0460; Beyotime, China) or Alexa Fluor 488 goat anti-rabbit IgG (catalog number A0423; Beyotime, China), for 30 min at 37 °C. The nuclei were stained with DAPI. The cells were imaged under fluorescence microscopy at 400 × magnification (Olympus BX-FLA, Japan)^45^.

### AD-mRFP-GFP-LC3 for autophagy detection

The FLSs were seeded into 12-well plates at a density of 1 × 10^5^ cells/well overnight. The MRFP-GFP-LC3 adenoviral vector stock, which was purchased from HanBio Technology Co. Ltd. (Shanghai, China), was adjusted to 50 MOI in 500 µL of culture medium. Then, the fibroblasts were incubated with adenoviruses for 2 h at 37 °C, and the culture medium was replaced with fresh medium containing 100 µg/mL aluminum particles. The cells were further cultured for 3 d. Autophagy was observed under fluorescence microscopy (Olympus BX-FLA, Japan) at 12, 24, 48, and 72 h, and the autophagic flux was evaluated by counting the number of GFP and mRFP puncta.

### TRAP staining

PBMCs were isolated from the peripheral blood of healthy male subjects and cultured in α-MEM/FBS supplemented with 25 ng/mL M-CSF and various 20%-conditioned media (CM) for 14–21 d. The culture medium and the supplemented factors were exchanged every 3 d for various CM. The CM were obtained from cultured FLSs under various conditions, including no aluminum particles + no LV-vector particles (Group A, negative control), aluminum particles (Group B, 100 µg/mL), aluminum particles + control LV-vector particles (Group C, 100 µg/mL), aluminum particles + LV-Beclin1 (Group D, 100 µg/mL), aluminum particles + LV-Beclin1-shRNA (Group E, 100 µg/mL), and aluminum particles + RANKL (Group F, 50 ng/mL, positive control). The cells and the sections of femoral samples were stained with tartrate-resistant acidic phosphatase (NO. 387, Sigma-Aldrich, USA) in accordance with the manufacturer’s instructions. The sections were then stained with or without hematoxylin^46^. TRAP-positive cells exhibiting three or more nuclei were considered osteoclasts and examined by light microscopy. These TRAP-positive cells (middle panel) revealed osteoclastic resorption pits on the bone disks.

### Real-time polymerase chain reaction

The mRNA was extracted from the cells and hip synovial tissues of patients using TRIzol reagent (Invitrogen, USA) in accordance with the manufacturer’s instructions. The gene expression level was determined by real-time polymerase chain reaction (RT-PCR) with SYBR Premix Ex Taq (Takara, Japan) and an ABI Prism 7500 sequence detection system (Applied Biosystems, USA). Actb/β-actin primers were used as internal controls. The primer sequence is listed in Table 1. The fold-change in the gene expression with respect to the control was calculated by the 2^-△△CT^ method.

**Table 1:** Primer sequences used for real-time PCR for human samples

Gene	Sequence (sense, antisense: 5′–3′)
MAP1LC3B	AGCAGCATCCAACCAAAATC
	CCGTTCACCAACAGGAAGAA
BECN1	TCCACAGAAAGTGCCAACAG
	GTCAAAAAGGTCCCCAGTGA
ATG5	GCCATCAATCGGAAACTCAT
	CAGCCACAGGACGAAACAG
SQSTM1(p62)	TAGGAACCCGCTACAAGTGC
	GAGAAGCCCTCAGACAGGTG
RANKL	GATGAAAGGAGGAAGCACCA
	TAAGGAGGGGTTGGAGACCT
RANK	GCCTTTCTCTTGGCATCATC
	TGCTTTTCCCCAGTATTTGC
OPG	CAGGCACTTGAGGCTTTCA
	TTGGGGTTTATTGGAGGAGA

### Western blot analysis

The cells and tissues were treated according to the study design and harvested at the specified time points. The samples were sonicated and lysed in RIPA lysis buffer with a protein inhibitor cocktail for 30 min on ice and centrifuged at 12,000x*g* for 10 min at 4 °C. Then, the total protein was collected, measured, and denatured. Approximately 60 µg of the total protein was subjected to SDS-PAGE and transferred to nitrocellulose membranes. The membranes were blocked with TBS (50 mM Tris, 200 mM NaCl, 0.2% Tween-20) and 5% nonfat milk powder for 2 h. The membranes were then incubated with the primary antibody overnight at 4 °C, followed by horseradish peroxidase (HRP)-conjugated anti-rabbit IgG or HRP-conjugated anti-mouse IgG antibodies (Beyotime, China) for 1 h. The membranes were detected for enhanced chemiluminescence (MultiSciences Biotech, China), and the resulting autoradiograms were quantified by densitometry with ImageJ software^47^.

### TEM and scanning electron microscopy (SEM)

Synovial tissues were collected from rTHA, THA, and femoral head replacement models in rats treated with aluminum particles for 8 weeks. The obtained synovial tissues were cut into 1–2 mm^3^ pieces. The FLSs were seeded in six-well plates at a density of 1 × 106 cells/well and treated with aluminum particles (100 μg/mL) for 48 h. Then, the tissues and the cells were fixed with 2.5% glutaraldehyde in 0.1 M sodium dihydrogen phosphate (pH 7.4) for 2 h and with 1% osmium tetroxide for 1 h at 4 °C, and then stained with 2% uranyl acetate in water for 1 h in the dark. After being hydrated with increasing concentrations of ethanol, the samples were embedded in epoxy resin and cut into 80 nm-thick sections. The sections were stained with uranyl acetate and lead citrate, and then observed with a transmission electron microscope (Philips CM, the Netherlands)^45,47^. The synovial samples were directly used after excision for SEM (QUANTA-250, Philips, Netherlands).

### Uncemented femoral components of rats

Ten 16-week-old SD rats weighing 400–450 g were scanned by a high-resolution microCT (SkyScan1172; SkyScan, Belgium) at a resolution of 10 µm. The parameters of the acetabulum, the femoral head, and the proximal femoral medullary cavity were measured and analyzed with a CT Analyzer (Version: 1.15.4.0). The diagrams of the uncemented femoral component were designed with CAD2014 software (version: 19.1.18.0) on the basis of the characteristics of the proximal femoral anatomical morphology. Double Medical Co., Ltd. (Xiamen, China) fabricated the femoral components, which consisted of a 15 mm and 130° neck shaft angle titanium alloy femoral prosthesis and a 3.8 mm cobalt–chromium–molybdenum alloy femoral head.

### Femoral head replacement model in rats

Sixteen-week-old SD rats weighing 400–450 g were used in our experiment. The animals were randomly divided into four groups: group A, femoral head replacement/ 50 µL PBS (control group); group B, femoral head replacement/aluminum particles (50 µL, 10 µg/mL); group C, femoral head replacement/aluminum particles (25 µL, 20 µg/mL)/Beclin1 (25 µL); and group D, femoral head replacement/aluminum particles (25 µL, 20 µg/mL)/LV-Beclin1-shRNA (25 µL). Groups A–D received the specified PBS, aluminum particles, and lentivirus vectors every 2 weeks through intra-articular hip injection until the animals were sacrificed.

The rats were anesthetized intraperitoneally with 1% sodium pentobarbital (50 mg/kg, body weight) and placed in prone position. The hibateral hind limb and half of the lower back of each animal was shaved and sterilized. A 2-cm-long vertical incision centered over the femoral head was made. The subcutaneous fascia was isolated, and the gluteus superficialis muscle was split in the direction of its muscle fibers by using vascular forceps. Then, the gluteus medius and the biceps femoris muscle were exposed and dissected along the muscle gap. The sciatic nerve was exposed and passed on the internal obturator muscle, which covered the posterior hip joint capsule. The capsule was opened with a T-shaped incision to expose the femoral head. The round ligament of the femur was cut, and the femoral head was dislocated. The head was removed. The piriformis fossa, which was located directly over the medullary canal, was the proper entry point. The medullary canal was gradually enlarged with a dentistry burr. Then, a cavity was drilled to a depth of 10 mm with a reamer, and the uncemented femoral component was placed in an approximately 20° anteversion. Finally, the hip stability, the leg length, and the soft tissue tension were assessed. The capsule was repaired, and the remaining tissue was sutured in layers. Buprenorphine was given for postoperative analgesia at a dose of 0.01–0.05 mg/kg (IP, SQ, q12 h), and cefazolin was administered at a dose of 20–40 mg/kg (IM, SQ, q daily) to prevent infection. For in vivo fluorescent labeling, six mice from each group were intraperitoneally injected with calcein (20 mg/kg body weight; Sigma-Aldrich, C0875) at day 12 and 2 days before being sacrificed.

### Statistical analysis

The results are presented as the mean ± standard error of at least three independent replications. Statistical analyses were performed using SPSS 16 statistical software (SPSS, Chicago, IL, USA). Multiple comparisons of data among the groups were analyzed by one-way ANOVA, followed by least significant difference testing (Fisher’s exact test). Comparisons between two groups were analyzed by Student’s *t*-test. A *P*-value of less than 0.05 was considered statistically significant.

## Electronic supplementary material

Figure S1

Figure S2

Figure S3

Figure S4

Figure S5

Captions for Supplementary figures
