# Supplementary figures and images for: Nano-sized Al2O3 particle-induced autophagy reduces osteolysis in aseptic loosening of total hip arthroplasty by negative feedback regulation of RANKL expression in fibroblasts
Source: Cell Death Dis. 2018 Aug 6;9(8):840. doi: 10.1038/s41419-018-0862-9 (PMC6079072; doi:10.1038/s41419-018-0862-9)

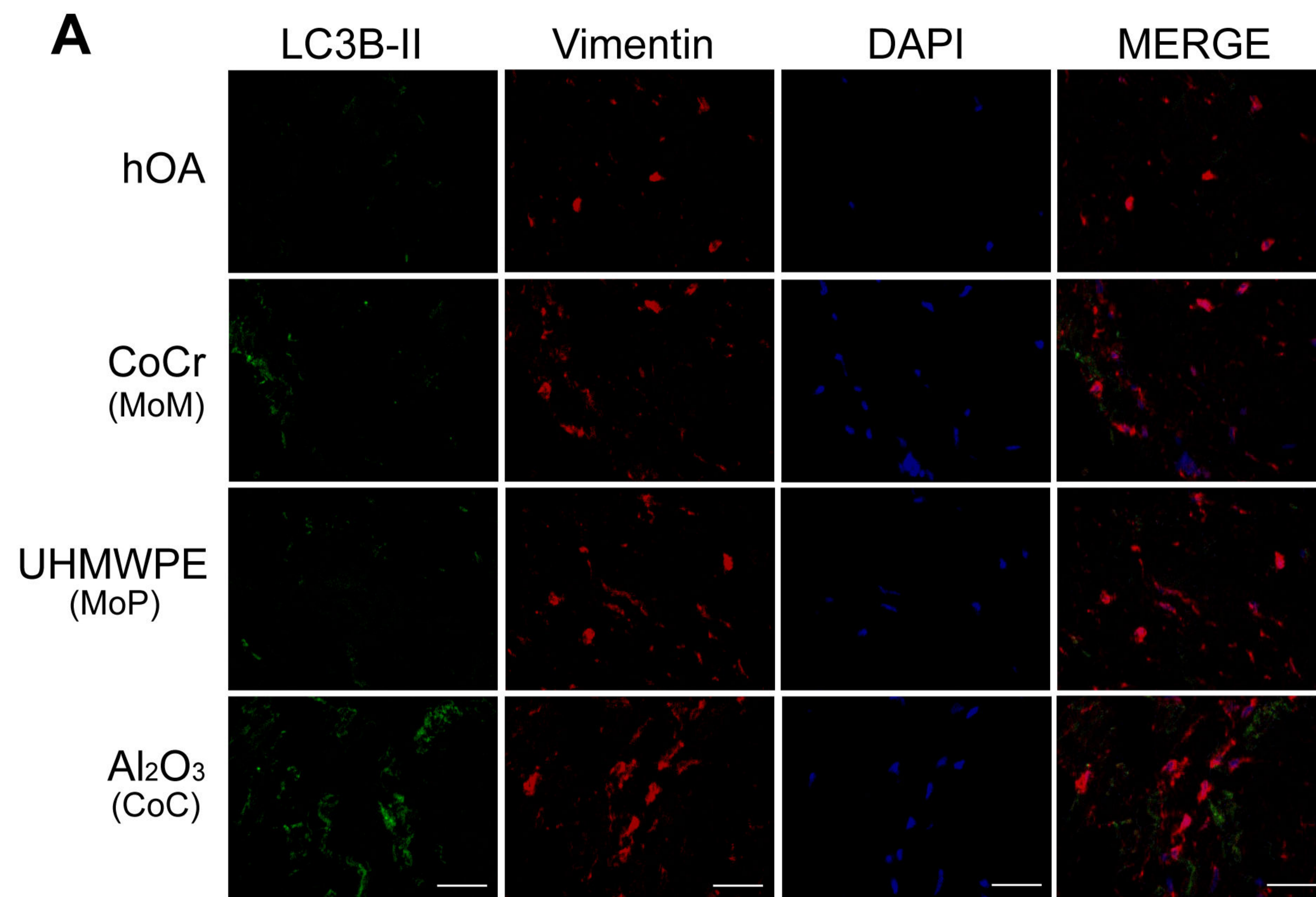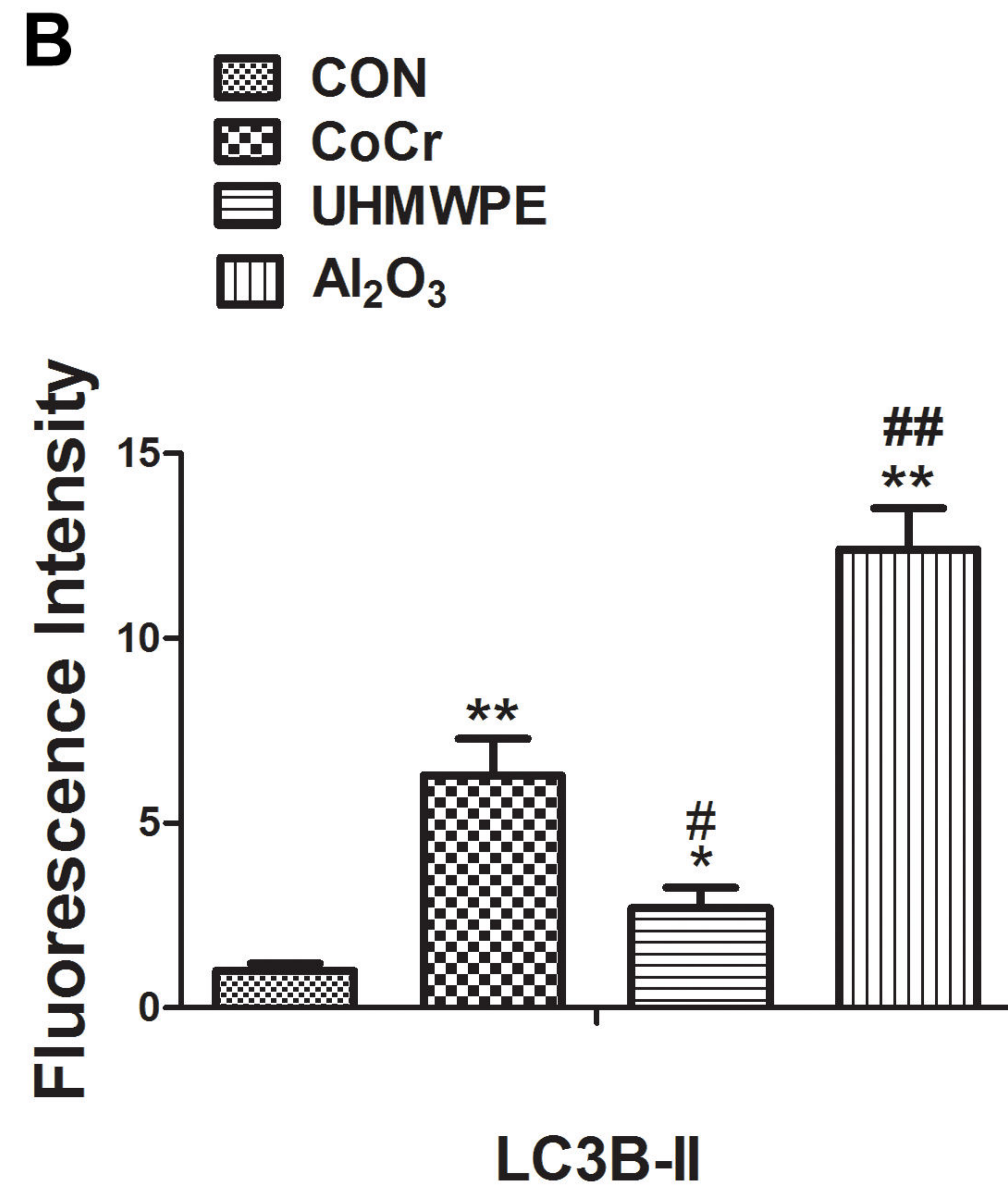

Supplement: Supplementary file 1 — Figure S1 [file 41419_2018_862_MOESM1_ESM.pdf]

**A**

**a**

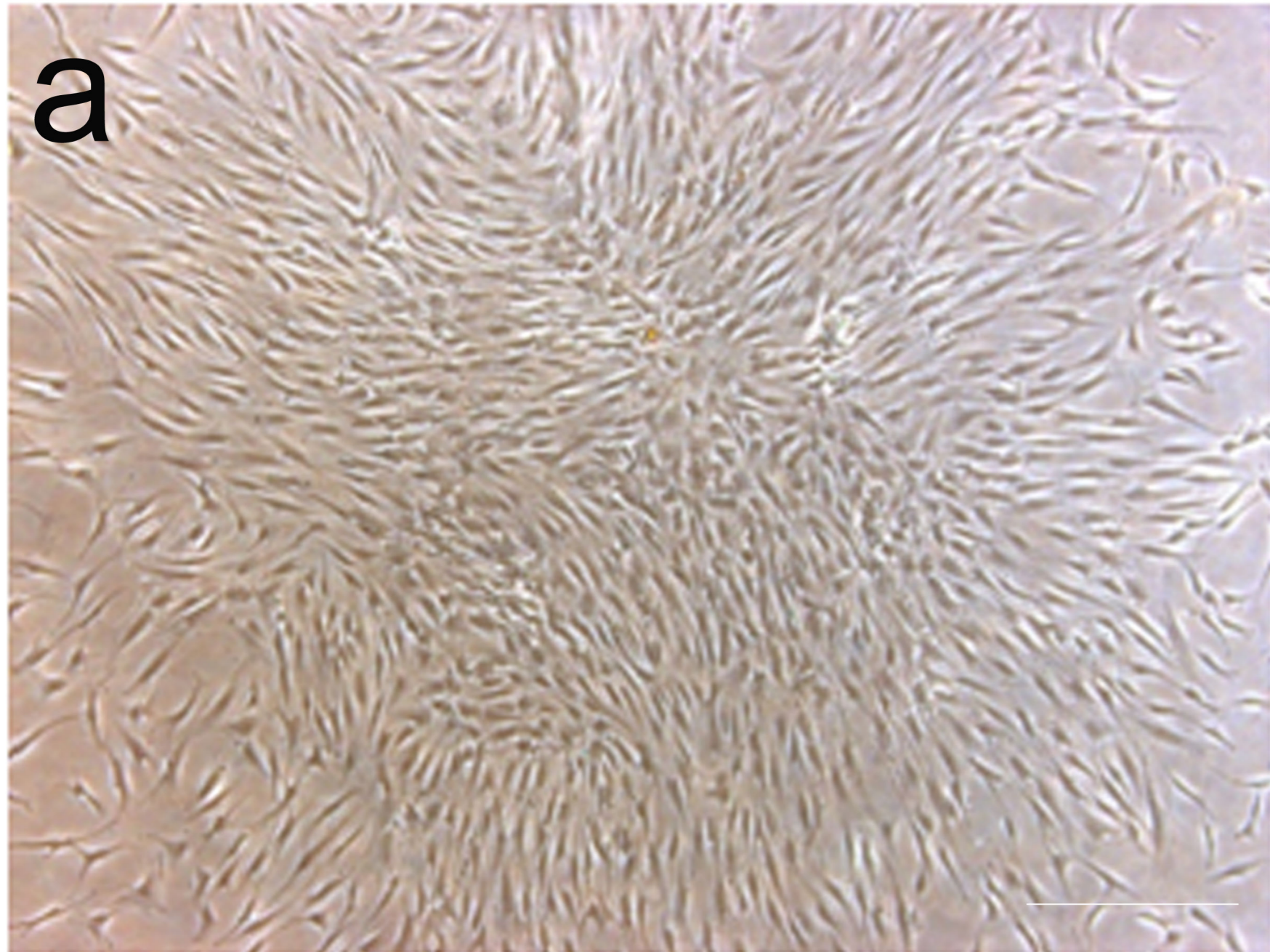

**b**

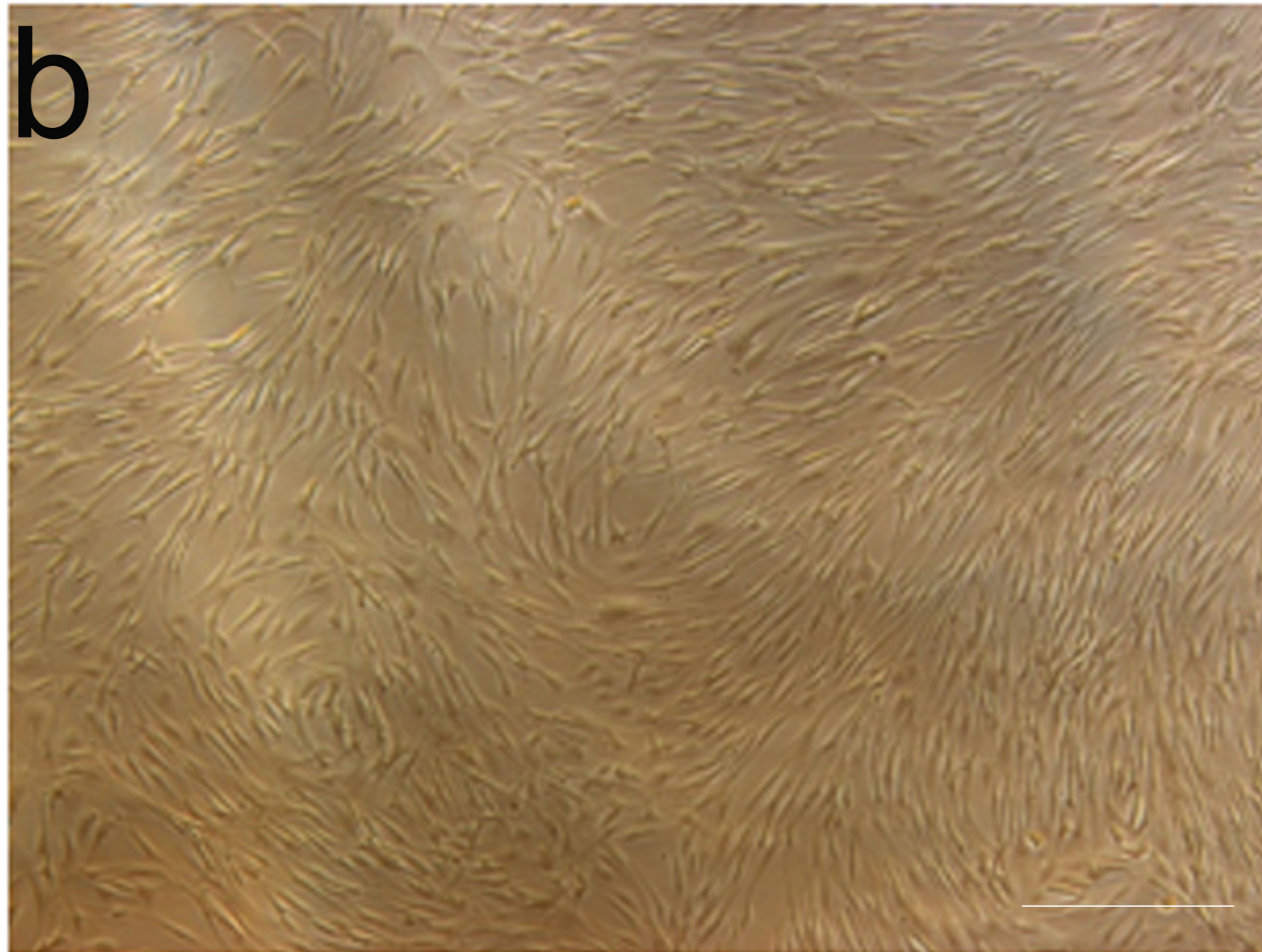

**c**

**Vimentin**

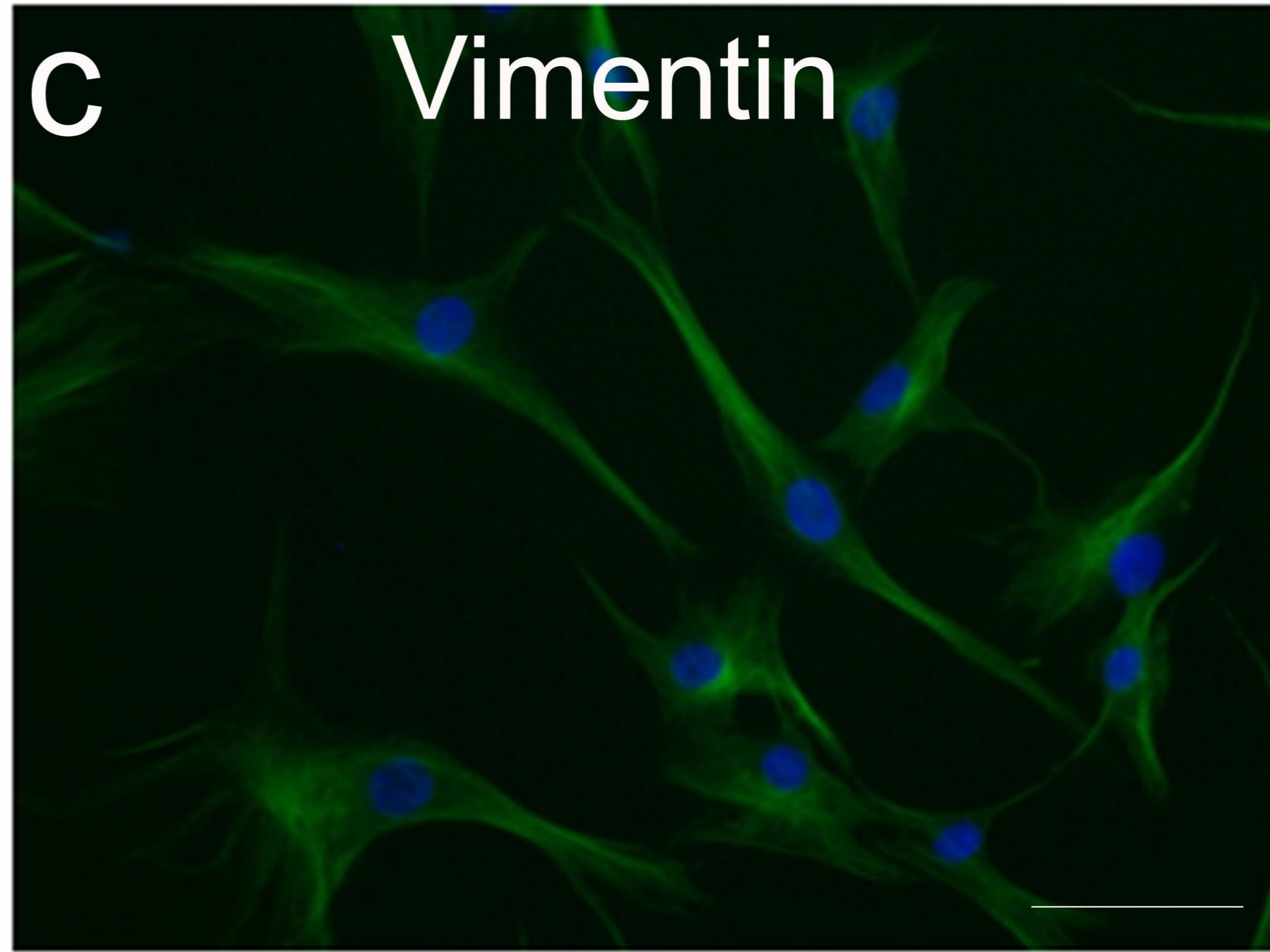

Supplement: Supplementary file 2 — Figure S2 [file 41419_2018_862_MOESM2_ESM.pdf]

**A**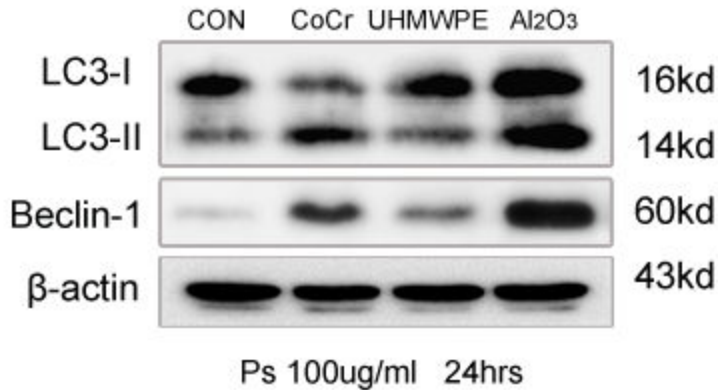**B**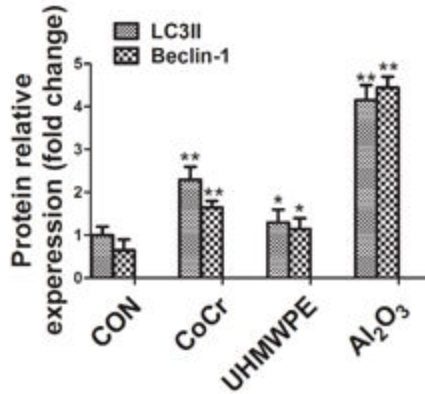

Supplement: Supplementary file 3 — Figure S3 [file 41419_2018_862_MOESM3_ESM.pdf]

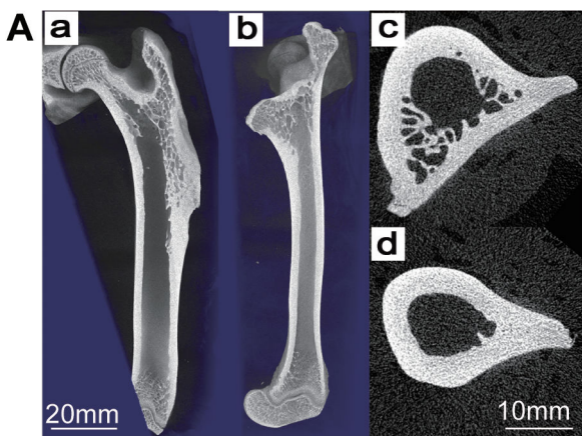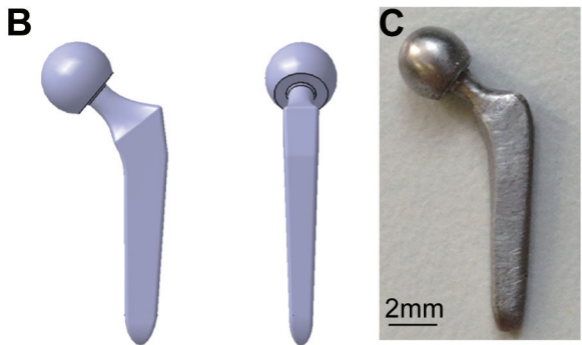

Supplement: Supplementary file 5 — Figure S5 [file 41419_2018_862_MOESM5_ESM.pdf]
