# Supplementary material for: Nano-sized Al2O3 particle-induced autophagy reduces osteolysis in aseptic loosening of total hip arthroplasty by negative feedback regulation of RANKL expression in fibroblasts
Source: Cell Death Dis. 2018 Aug 6;9(8):840. doi: 10.1038/s41419-018-0862-9 (PMC6079072; doi:10.1038/s41419-018-0862-9)
Supplement: Supplementary file 4 — Figure S4 [file 41419_2018_862_MOESM4_ESM.pdf]

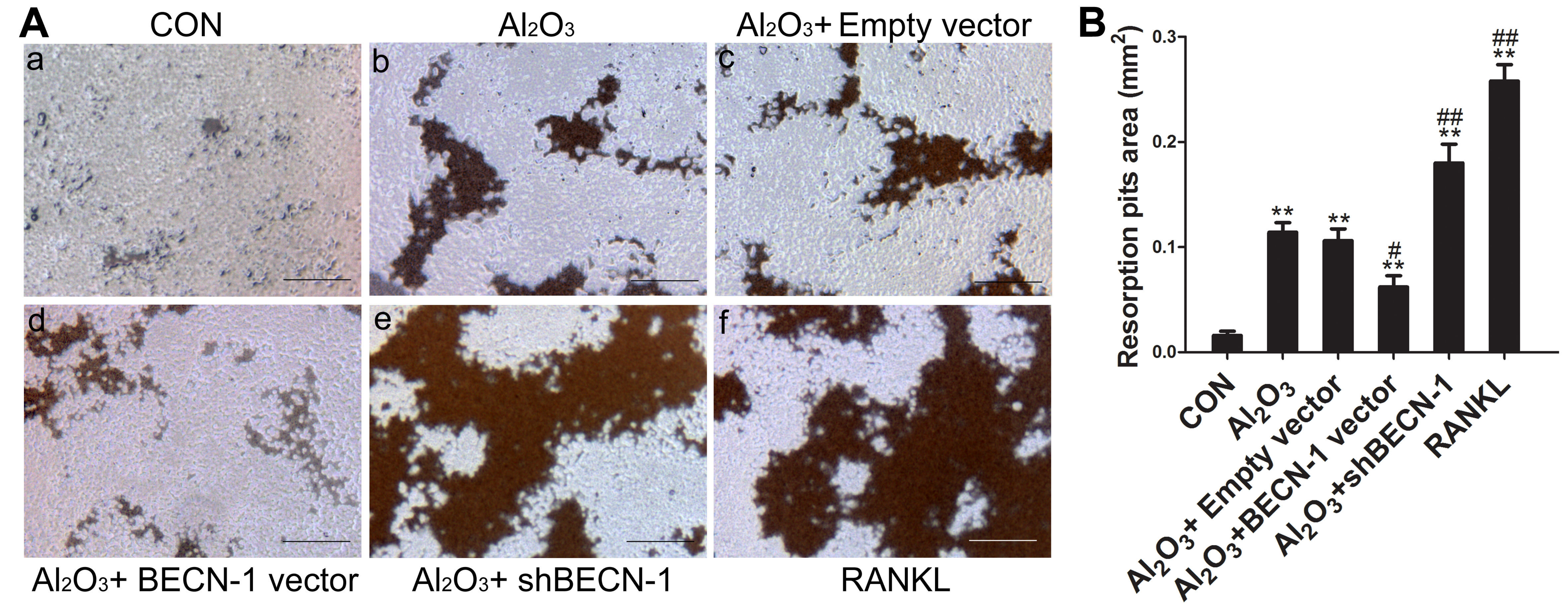

A
CON
Al_2O_3
Al_2O_3+ Empty vector
a
b
c
d
e
f
Al_2O_3+ BECN-1 vector
Al_2O_3+ shBECN-1
RANKL
B
Resorption pits area (mm^2)
0.0
0.1
0.2
0.3
**
**
#
**
##
**
##
**
CON
Al_2O_3
Al_2O_3+ Empty vector
Al_2O_3+BECN-1 vector
Al_2O_3+shBECN-1
RANKL
