# Supplementary material for: Nano-sized Al2O3 particle-induced autophagy reduces osteolysis in aseptic loosening of total hip arthroplasty by negative feedback regulation of RANKL expression in fibroblasts
Source: Cell Death Dis. 2018 Aug 6;9(8):840. doi: 10.1038/s41419-018-0862-9 (PMC6079072; doi:10.1038/s41419-018-0862-9)
Supplement: Supplementary file 6 — Captions for Supplementary figures [file 41419_2018_862_MOESM6_ESM.docx]

**Figure** S1 Autophagy in the synovial tissue of rTHA.

1. Immunofluorescence staining was performed to determine the expression of

LC3B-Ⅱin the synovial tissue of hip joints from the patients with rTHA (MoM, MoP and CoC) and hOA. Nucleus (DAPI, blue), LC3B-Ⅱ(green), and Vimentin (red). Scale
bar=25µm. Quantification of the fluorescence intensity of LC3 was presented in (B). ( * compared with the hOA group, *P < 0.05, **P < 0.01; # compared with the MoM group , #P < 0.05, ##P < 0.01).

**Figure** S2 Culture and identification of FLSs

(A)FLSs by light microscopy, a(Primary), b(P1), c FLSs were identified using immunofluorescence for vimentin. Nucleus (DAPI, blue), Vimentin (green). (a,b Scale
bar=200µm; c Scale bar=25µm).

**Figure** S3 Nano-Ps induced autophagy in the fibroblasts.

(A) Western blots were performed after FLSs were incubated with the different Nano-Ps (100 µg/ml) for 24 h. (B) The density of the western blots bands presented in (A) was quantified using Image J software. (n=3, * compared with the hOA group, *P < 0.05, **P < 0.01; # compared with the MoM group , #P < 0.05, ##P < 0.01).

**Figure** S4 Visualization of resorption pits from human osteoclast precursor cells.

(A) PBMCs were cultured on the Corning Osteo Assay Surface in α-MEM/FBS supplemented with 25 ng/mL M-CSF and various 20% conditioned media (CM) for 14–21 d. The CM were obtained as Materials and methods (trap staining). So human osteoclast precursor cells were formed. Scale bar=100µm. (B) Data are expressed as mean ± SD of three independent experiments. (* compared with Control group, *P < 0.05, **P < 0.01; # compared with Empty vector , # P<0.05, ##P<0.01)

**Figure** S5 Design of biologic femoral head prosthesis of rat.

(A) Micro-CT were used to measure the parameters of the acetabular, femoral head, proximal femoral medulla of the 16w SD rats. (a,b Scale bar=20mm; c,d Scale bar=10mm). (B,C）The schematic drawing and 3D printing object of the femoral head prosthesis. Scale
Scale bar=2mm.
